# Binocular video head impulse test: Normative data study

**DOI:** 10.3389/fneur.2023.1153102

**Published:** 2023-05-03

**Authors:** Maja Striteska, Martin Chovanec, Tobias Steinmetzer, Viktor Chrobok, Oliver Profant, Erich Schneider, Jan Kremlacek, Martin Valis

**Affiliations:** ^1^Department of Otorhinolaryngology and Head and Neck Surgery, University Hospital Hradec Kralove, Charles University, Faculty of Medicine in Hradec Kralove, Hradec Kralove, Czechia; ^2^Department of Otorhinolaryngology, Third Faculty of Medicine, Charles University and University Hospital Kralovske Vinohrady, Prague, Czechia; ^3^Institute of Medical Technology, Brandenburg University of Technology Cottbus-Senftenberg, Cottbus, Germany; ^4^Department of Auditory Neuroscience, Institute of Experimental Medicine (ASCR), Prague, Czechia; ^5^Department of Medical Biophysics, Faculty of Medicine in Hradec Králové, Charles University, Hradec Kralove, Czechia; ^6^Department of Neurology, University Hospital Hradec Kralove, Charles University, Faculty of Medicine in Hradec Kralove, Prague, Czechia

**Keywords:** binocular video head impulse test, conjugate gaze, adduction, abduction, ductional VOR asymmetry index, dysconjugacy ratio, monocular VOR asymmetry index, vestibuloocular reflex

## Abstract

**Introduction:**

The video head impulse test (vHIT) evaluates the vestibulo-ocular reflex (VOR). It’s usually recorded from only one eye. Newer vHIT devices allow a binocular quantification of the VOR.

**Purpose (Aim):**

To investigate the advantages of simultaneously recorded binocular vHIT (bvHIT) to detect the differences between the VOR gains of the adducting and the abducting eye, to define the most precise VOR measure, and to assess gaze dys/conjugacy. We aimed to establish normative values for bvHIT adducting/abducting eye VOR gains and to introduce the VOR dysconjugacy ratio (vorDR) between adducting and abducting eyes for bvHIT.

**Methods:**

We enrolled 44 healthy adult participants in a cross-sectional, prospective study using a repeated-measures design to assess test–retest reliability. A binocular EyeSeeCam Sci 2 device was used to simultaneously record bvHIT from both eyes during impulsive head stimulation in the horizontal plane.

**Results:**

Pooled bvHIT retest gains of the adducting eye significantly exceeded those of the abducting eye (mean (SD): 1.08 (SD = 0.06), 0.95 (SD = 0.06), respectively). Both adduction and abduction gains showed similar variability, suggesting comparable precision and therefore equal suitability for VOR asymmetry assessment. The pooled vorDR here introduced to bvHIT was 1.13 (SD = 0.05). The test–retest repeatability coefficient was 0.06.

**Conclusion:**

Our study provides normative values reflecting the conjugacy of eye movement responses to horizontal bvHIT in healthy participants. The results were similar to a previous study using the gold-standard scleral search coil, which also reported greater VOR gains in the adducting than in the abducting eye. In analogy to the analysis of saccade conjugacy, we propose the use of a novel bvHIT dysconjugacy ratio to assess dys/conjugacy of VOR-induced eye movements. In addition, to accurately assess VOR asymmetry, and to avoid directional gain preponderance between adduction and abduction VOR-induced eye movements leading to monocular vHIT bias, we recommend using a binocular ductional VOR asymmetry index that compares the VOR gains of only the abduction or only the adduction movements of both eyes.

## Introduction

Accurate control of binocular eye movements is essential to direct the fovea of each eye at an object in the visual field. During locomotion, visual exploration requires coordination between gaze-stabilizing reflexes and gaze-shifting eye movements to ensure clear vision and depth perception. Failure of either system, or failure to achieve binocular coordination, results in blurred vision, diplopia, and loss of stereo acuity (1, 2).

The reflex that stabilizes gaze on a target, for example, during locomotion, by rotating the eyes in the opposite direction to head movement is the vestibulo-ocular reflex (VOR). The traditional measure of angular VOR function is gain, defined as the ratio of eye and head angular velocity.

Depending on the distance and eccentricity of the visual target during VOR-induced eye movements, the two eyes’ lines of sight should be parallel when viewing distant objects (conjugate gaze) or intersect (converge) at the location of a near target. VOR gain increases as the fixated target moves closer to the observer (3–5), reflecting the interaction between the version and vergence systems in the VOR.

The video head impulse test (vHIT) directly quantifies VOR function by assessing VOR gain, and it objectively detects both covert and overt refixation saccades as an indirect sign of canal paresis. To date, the vHIT has mostly been used monocularly. Binocular vHIT would allow simultaneous recording of the movements of both eyes resulting from VOR activation by a head impulse. Importantly, the nasal movement of the ADducting (AD) eye and the temporal movement of the ABducting (AB) eye could be analyzed separately. The need for an accurate binocular head impulse test was highlighted in 2008 in a study using a gold standard scleral search coil to measure head and binocular eye movements (6). The study showed that the difference between the gains of the adducting and abducting eye reached 15.3% at head accelerations greater than 3,234 ^o^/s^2^ (6). However, when only abduction gains were compared between both eyes, VOR symmetry was stable across all head accelerations. While accurate VOR measurement is a prerequisite for the diagnosis of unilateral vestibular loss, the disadvantage of using a monocular vHIT system is a directional gain preponderance of adduction over abduction VOR eye movement responses (6, 7). Therefore, it is crucial to minimize this bias, for example, by calculating the adduction- or abduction-related gains from the binocular recordings. Based on the lower variability, the search coil study recommended analysis of the VOR gains of the abducting eyes to obtain directional symmetry of VOR gain measurements in normal subjects.

### Study aims

Although these findings highlighted the need for binocular vHIT, normative ranges for binocular vHIT (bvHIT) have not yet been established.

The HIT has the potential to assess not only peripheral vestibular function by evaluating the VOR gain response but also the complete VOR arc with its nuclear, internuclear, and infranuclear pathways, including the oculomotor nerves and muscles. Simultaneous binocular recording adds the ability to assess the central pathways by comparing centrally controlled conjugate eye movements between the adducting and abducting eyes. Our study aimed to establish normative ranges for adduction-and abduction-related VOR gains and to introduce a dysconjugacy ratio (vorDR) (8) between the two.

## Methods

### Participants

We measured 44 healthy adults (22 male, 22 female, 20 to 70 years of age, mean age 35, SD 12.5) in a prospective cross-sectional study using a repeated-measures design.

### Standard protocol approvals, registrations, and patient consent

Before including a participant in this study, we received their written informed consent. The protocol was approved by a local ethics committee and was in accordance with the Declaration of Helsinki (Reference number 202106 P08). The inclusion criterion was a negative history of any balance disturbance or of any oculomotor deficit due to an underlying neurological condition.

### Study device

**Figure 1:**
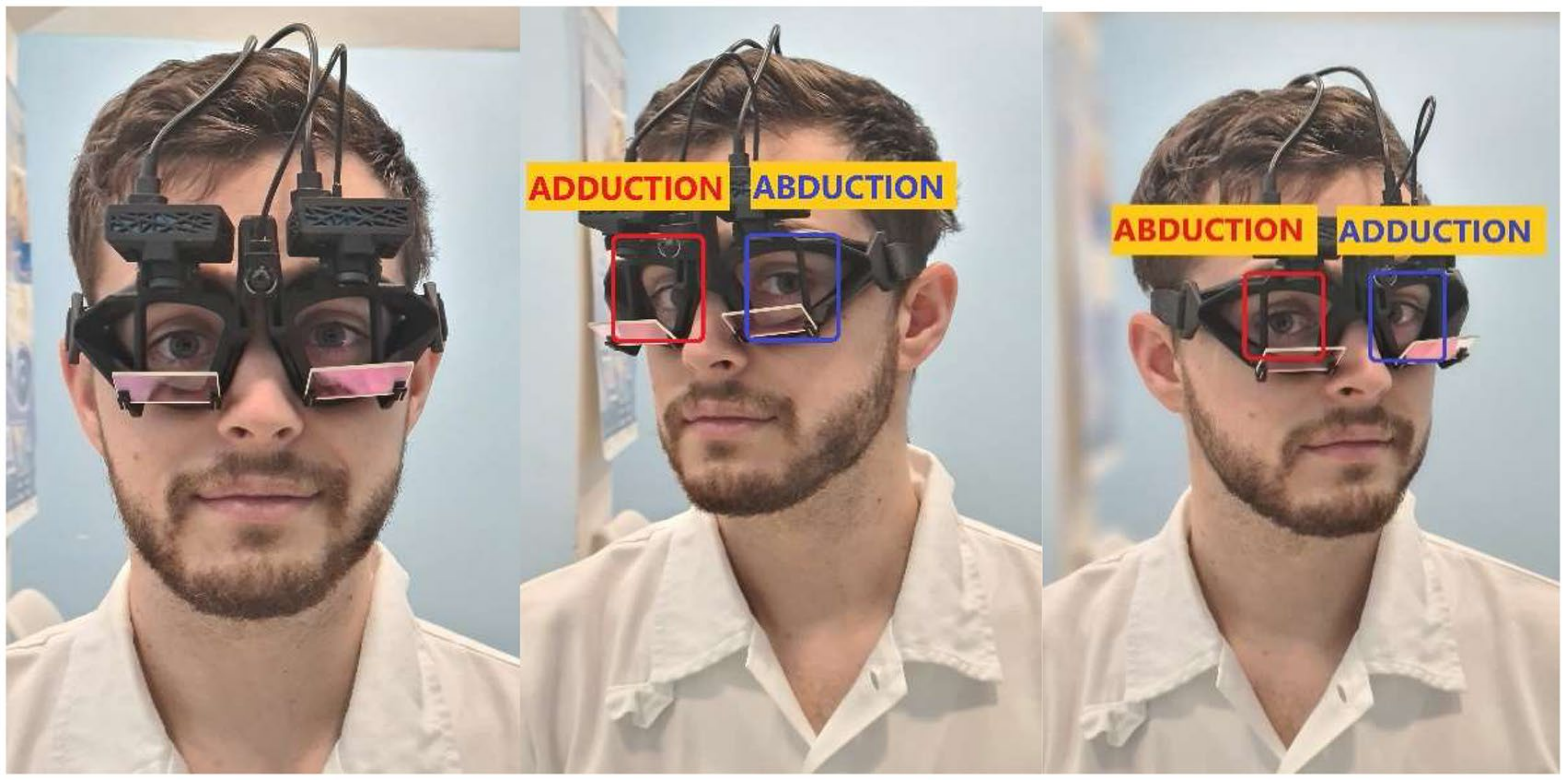
Binocular vHIT (EyeSeeTec Sci 2): Two high-speed cameras are attached to tightly fitting goggles. Note the ductions of the right (red) and left (blue) eyes during rightward and leftward impulses.

For simultaneous recordings of head and binocular eye movements, we used a binocular EyeSeeCam Sci 2 device (EyeSeeTec, Munich, Germany) (Figure 1). The study device is a successor version of the previous EyeSeeCam Sci 1 and Interacoustics EyeSeeCam vHIT (Interacoustics, Middelfart, Denmark) systems. Attached to the new goggles, there was a pair of synchronized high-speed cameras which tracked the pupil to determine eye position at sampling rates of either 500 Hz or 250 Hz. For this study we used the lower sampling rate of 250 Hz. An inertial measurement unit integrated into the left camera measured angular head velocity at the same sampling rate.

### Study methods

Participants were seated 3 meters in front of a fixation dot on a white wall. The fixation dot was black, it contained two lines crossing at the center, and had a diameter of 5 cm, which provided a good fixation target also for myopic participants. The target distance of 3 m was chosen to minimize the effect of vergence on VOR gain, as it is well known that VOR gain increases with decreasing target distance (3, 9) and that this effect vanishes at distances of more than 2 meters (4). First, the study device was calibrated with the participants sequentially fixating five laser dots on the wall 3 meters in front of them. The dots were projected from a goggle-mounted laser and a diffraction grating. After calibration, 14 horizontal head impulses were completed (7 to both sides) during each test. The examiner grasped the head of a participant from behind and moved it briskly from center to each side with unpredictable timing and direction, aiming at an angular displacement amplitude of 20^o^ and a peak velocity in the range of 150^o^ to 250^o^/s. To assess test–retest reliability, the sequence consisting of calibration and seven impulses in both the left and right directions was repeated a second time by the same examiner (MS), who had a seven-year clinical experience in using vHIT. The sequence was repeated immediately if any technical error was noted. Test–retest was applied to all participants within one session to avoid biases caused by changes in their health status.

### Invalid impulses, artifacts, goggle slippage

The proprietary algorithm classified impulses as valid if no eye blinks or other artifacts were detected. Invalid impulses were discarded from the analysis. The remaining valid impulses were subsequently inspected visually for remaining artifacts. Impulses with artifacts not detected automatically were manually removed using the interactive Traces Editor of the EyeSeeCam Sci 2 software. Only goggle slippage or pupil detection artifacts, but not VOR gain or the presence of corrective saccades, were used as criteria to remove impulses. On average, 6.5 (range four to seven) out of seven impulses per measurement were considered valid to remain in the data set for analysis. The recordings of five subjects were excluded from the study due to an insufficient number (less than four) of valid impulses without artifacts. Thirty-nine subjects were used in the data set (20 male, 19 female, mean age 36, SD 13).

### Metrics

Our study used vHIT gains, dysconjugacy ratios, and asymmetry indexes as continuous quantitative metrics. We analyzed the binocular results of three different vHIT gain calculation methods reported by the EyeSeeCam Sci 2 system: (1) Regression gain (10, 11); (2) Instantaneous gain at 60 ms (10, 11); (3) Median gain 0–100 ms calculated as the median of the ratios of eye and head velocity medians in a window between 0 and 100 ms. For all metrics, the EyeSeeCam Sci software also reported the standard deviations (SD) calculated from the four to seven valid stimulations.

Furthermore, the question of how conjugate and symmetrical the VOR eye movements are was addressed by deriving further ratios and indexes from the reported gain values for both the left and right eyes as well as for both leftward and rightward head impulse directions. Asymmetry indices were calculated in analogy to previous definitions of VOR asymmetry indices (6, 9). Abbreviations used in the succeeding equations are defined in the contingency table in Table 1.

**Table 1:** Contingency table mapping leftward and rightward impulse directions as well as left and right eyes to color-coded abbreviations for AD- and ABduction.

		Impulse direction	
		Rightward	Leftward
Eye	Right (RE)	AD_RE_	AB_RE_
	Left (LE)	AB_LE_	AD_LE_

During the rightward impulse, the right eye (red) ADducts, and the left eye (blue) ABducts (in the “Rightward” column) to follow the target. During the leftward impulse, the right eye (red) ABducts, and the left eye (blue) ADducts.

#### Monocular VOR asymmetry index

**Figure 2:**
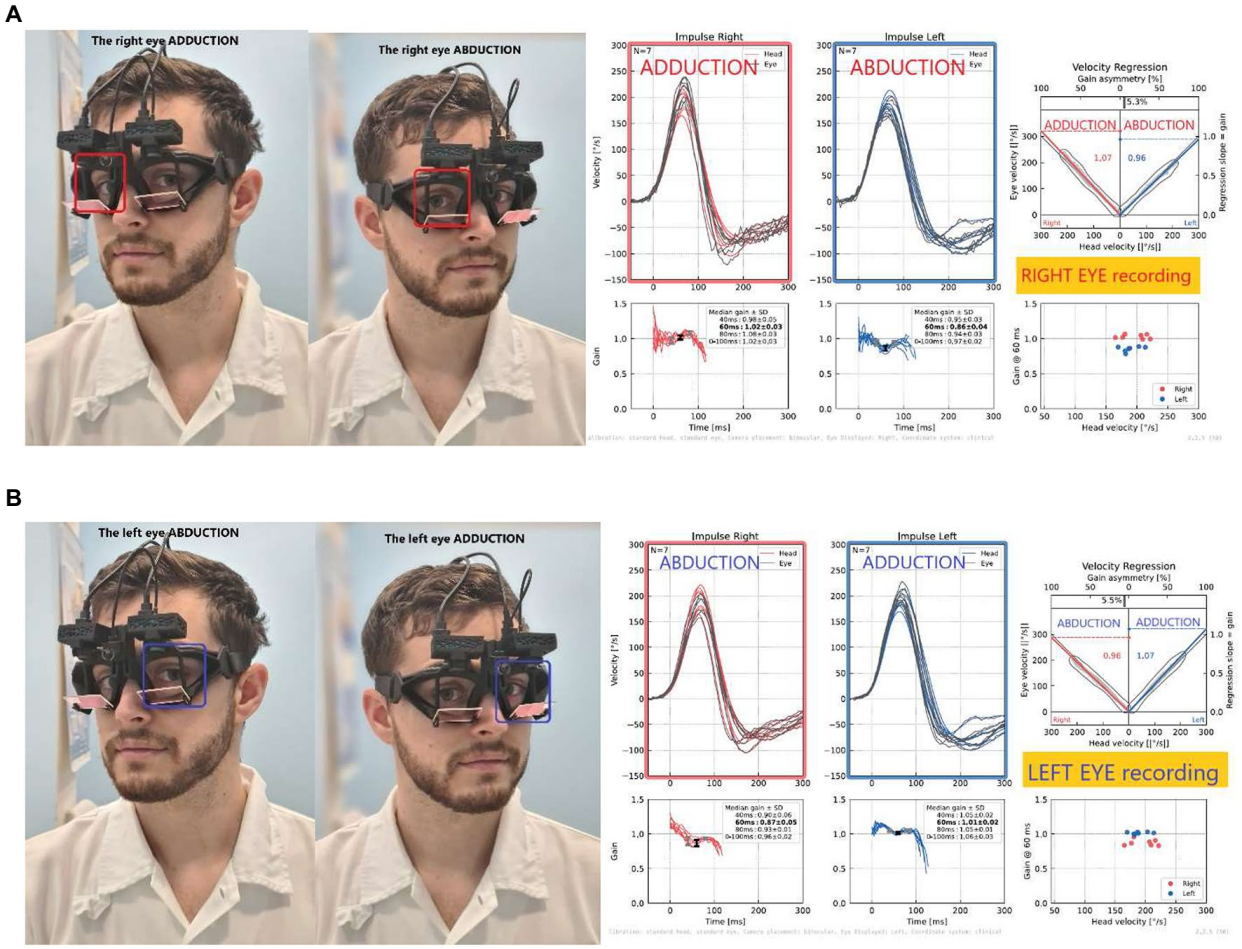
Monocular recordings from the right **(A)** and left **(B)** eyes: Note the higher gain during right impulse on the right ADducting eye **(A)**, while opposite on the left eye **(B)**, demonstrating the directional gain preponderance when recorded only from one eye (left or right). Monocular VOR asymmetry calculated for the right eye; in this case, RE_m-vorAI was 5% asymmetry, the same for the left eye.

To assess the possible directional gain preponderance between AD and AB eyes, we first evaluated the monocular recordings of each of the two cameras separately. As most head-mounted vHIT devices provide only one camera, clinicians are familiar with the evaluation of such monocular recordings. We compared left- and rightward impulse gains monocularly analyzed from each eye to evaluate monocular VOR asymmetry (Figure 2). We calculated the monocular VOR asymmetry index (m-vorAI) by using Eq. 1 and the abbreviations from Table 1. The equation contains the absolute value of the difference between gains in the numerator.

Eq. 1:

(a) Right Eye (RE) Monocular VOR asymmetry:
  RE_m-vorAI = | AD_RE_ − AB_RE_ | / ( AD_RE_ + AB_RE_ ) × 100%.
(b) Left Eye (LE) Monocular VOR asymmetry:
  LE_m-vorAI = | AD_LE_ - AB_LE_ | / ( AD_LE_ + AB_LE_ ) × 100%.

#### Ductional VOR asymmetry index

**Figure 3:**
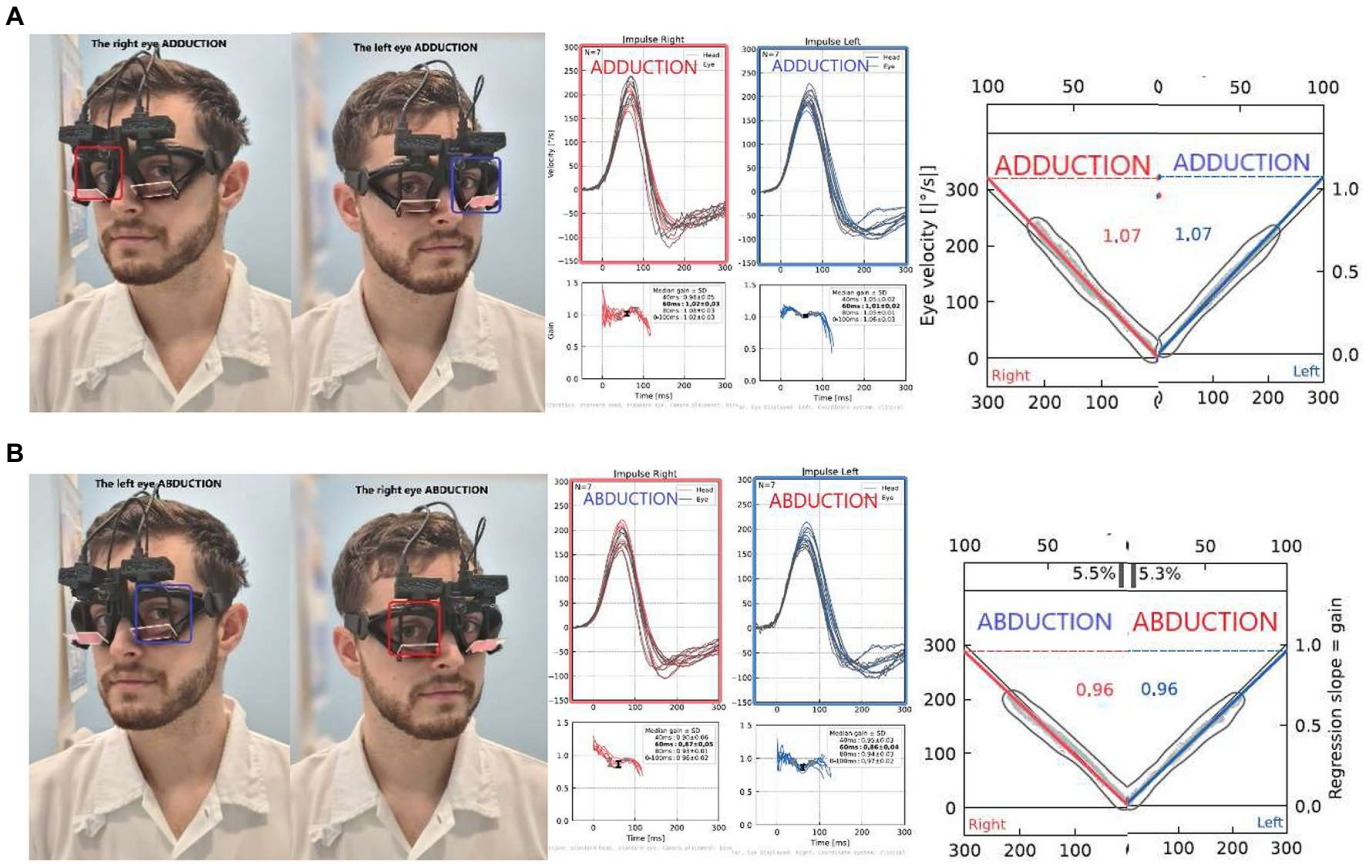
Ductional VOR asymmetry of one participant: Comparison of left and right impulses recorded from only ADducting **(A)** or ABducting **(B)** eyes: In this case, symmetrical VOR responses can be observed, calculated as 0% asymmetry for both **(A)** AD gains (AD vorDAI = 0%), as well as **(B)** AB gains (AB vorDAI = 0%).

To avoid the effects of directional gain preponderance on VOR asymmetry typically obtained from monocular recordings (6, 7), and to assess the most precise VOR asymmetry metric, we computed ductional VOR asymmetry indices (vorDAI) separately for ADduction and ABduction eye movement responses to left- and rightward head impulses. Specifically, the ADduction asymmetry is calculated from only adducting eyes during impulsive testing: rightward impulses from the right eye and leftward impulses from the left eye (Figure 3), and vice versa for ABduction (calculated from both ABducting eyes). The vorDAI were calculated using the abbreviations from Table 1 in Eq. 2, which contains the absolute value of the difference between ductional gains in the numerator:

Eq. 2:

(a) ADduction VOR asymmetry index:
  AD_vorDAI = | AD_RE_ − AD_LE_ | / ( AD_RE_ + AD_LE_ ) × 100%.
(b) ABduction VOR asymmetry index:
  AB_vorDAI = | AB_RE_ − AB_LE_ | / ( AB_RE_ + AB_LE_ ) × 100%.

#### Binocular vHIT dysconjugacy ratio

**Figure 4:**
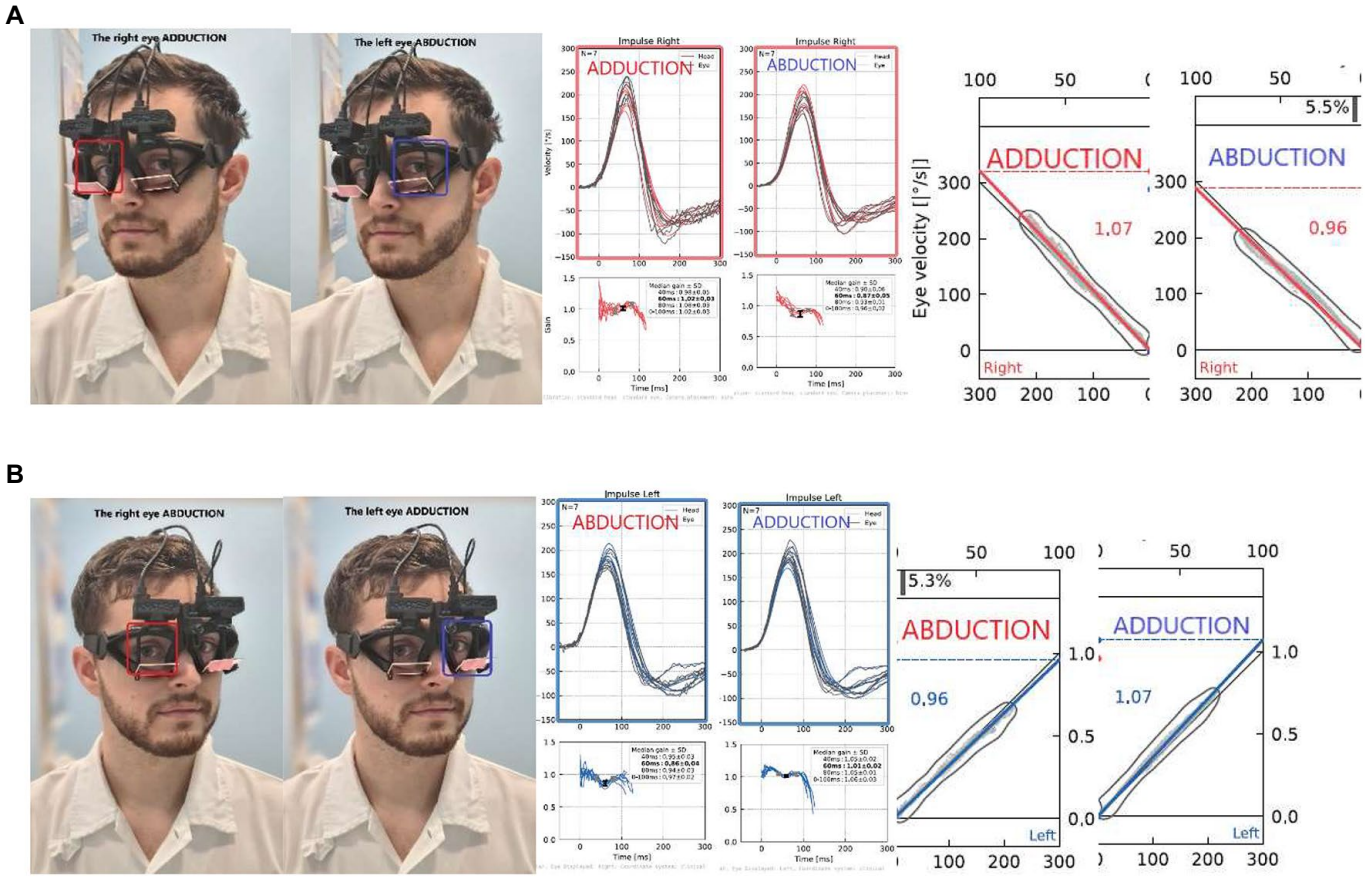
Dysconjugacy ratio (vorDR) of leftward gaze during rightward impulse **(A)** and rightward gaze during leftward impulse **(B)**. The calculation is based on the direction of an impulse: In this exemplary case, the **(A)** rightward dysconjugacy ratio (Rightward vorDR) is 1.07/0.96 = 1.11, and the same result is calculated for **(B)** leftward dysconjugacy ratio (Leftward vorDR) is 1.07/0.96 = 1.11.

To assess eye movement dysconjugacy and to evaluate the contribution of central inter- and infra-nuclear oculomotor pathways to the execution of vestibularly-induced conjugate eye movements, we compared the same direction impulses recorded simultaneously from both eyes. For example, during a leftward head impulse, we measured the adduction response of the left eye, and the abduction response of the right eye (Figure 4). To quantify eye movement dys/conjugacy during impulsive testing, we propose the use of a bvHIT dysconjugacy ratio (vorDR) between ADducting and Abducting eyes during the same direction impulse recorded from both eyes, as previously suggested in the literature (8). We defined the vorDR for bvHIT such that ADduction is in the numerator, giving a value >1 when the ADduction (AD) gain is greater than the ABduction (AB) gain. The calculation is based on the direction of an impulse, which allows the assessment of both unidirectional dysconjugacy, such as an isolated oculomotor deficit in unilateral INO, or bidirectional dysconjugacy, such as bilateral INO.

Eq. 3:

(a) Rightward impulse dysconjugacy ratio:
  Rightward_vorDR = AD_RE_ / AB_LE_ .
(b) Leftward impulse dysconjugacy ratio:
  Leftward_vorDR = AD_LE_ / AB_RE_.

### Statistical analysis

A repeated-measures study design with three within factors, each with two levels, was used: duction (ABduction, ADduction), eye (left, right), and repetition (test, retest). Continuous normally distributed data are reported as mean (SD), with standard deviation in parentheses. Indices are reported as median (IQR), with interquartile range in parentheses. Statistical computations were conducted with JASP (JASP Team, 2022), Python (Version 3.9) with Pandas (Version 1.3.2), and R (R Core Team, 2022). All metrics were tested for normality by visual inspection of qq plots and subsequent Shapiro–Wilk testing. Levene’s test was used to verify variance homogeneity. Differences and interactions in VOR gains between factor levels were assessed with a repeated measures analysis of variance (ANOVA). Both frequentist and Bayesian analyzes were performed for repeated measures ANOVA. For frequentist analyzes, *p* < 0.05 was considered statistically significant. Separate tests were performed for the metrics instantaneous gain, median gain, and regression gain.

The distributions of gains as well as derived dysconjugacy ratios and asymmetry indexes were assessed visually using qq plots and tested for normality using Shapiro-Wilks tests. F-tests of within-subject SD were used to answer the questions of (1) which gain calculation method, (2) which duction level (ABduction, ADduction), and (3) which repetition level (test, retest) yielded the better precision for future use in bvHIT.

## Results

### Normative values

**Figure 5:**
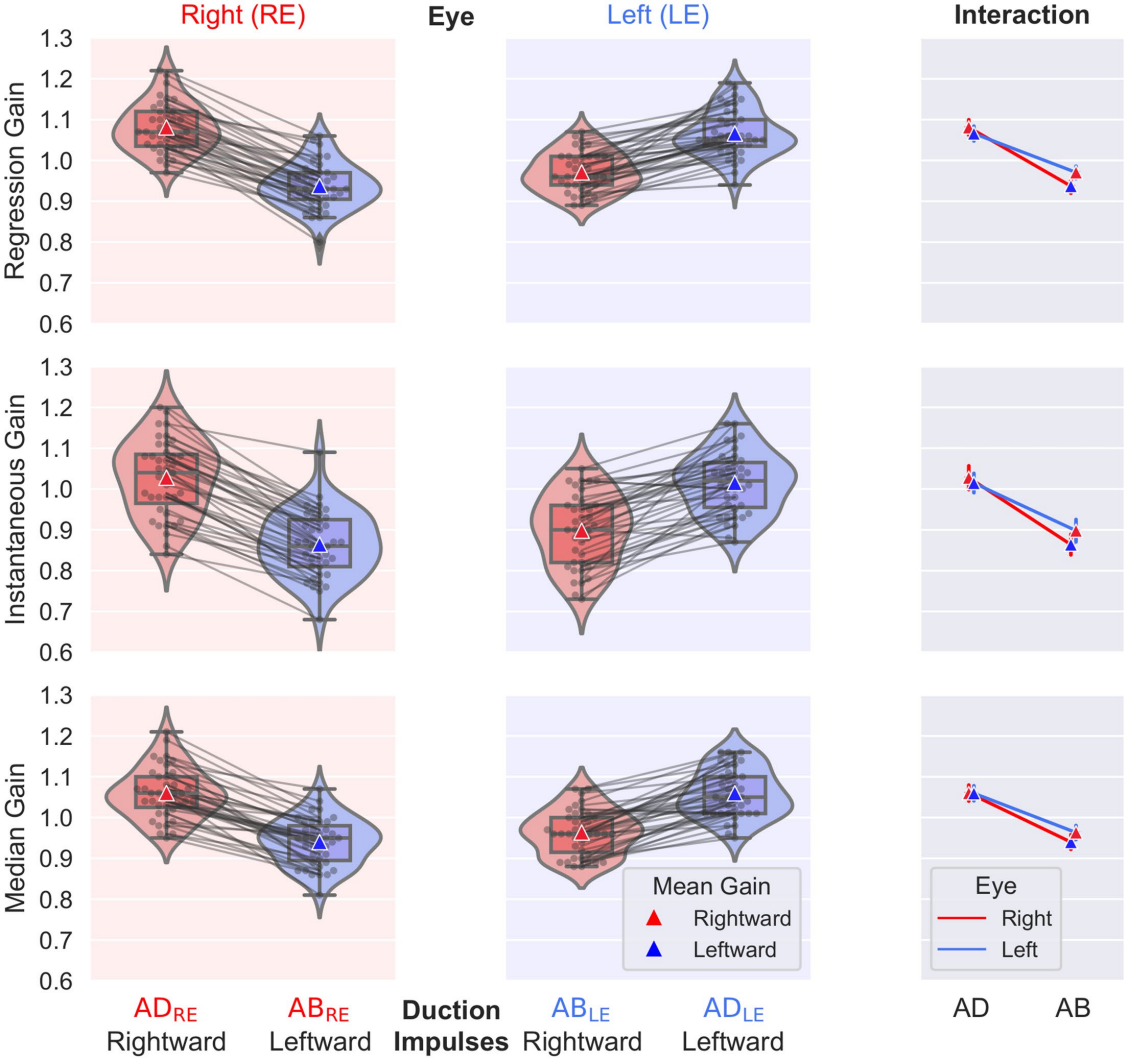
Individual gain values, box plots of descriptive statistics, and distributions for the factors eye (left, right) and duction (abduction, adduction). The results of the regression gain (top), instantaneous gain (middle), and median gain (bottom) calculations are shown. The interaction plot on the right presents the interaction between the factors eye and duction.

The normative values and ranges for the regression gain, instantaneous gain, and median gain metrics, and bvHIT dysconjugacy ratio are shown in Table 2. Normative values and ranges for the monocular and ductional VOR asymmetry indices are shown in Table 3. The gain and ratio metrics were normally distributed (*W* = 0.95, *p* > 0.08), and their between-subject SDs were F-distributed. Nonparametric normative values are reported for the VOR asymmetry indices. The distributions of the three different gain metrics can be visually assessed from Figure 5. Levene’s test indicated equality of variances [*F* (7,304) = 0.66, *p* > 0.71].

**Table 2:** Normative values and ranges for the three VOR gain methods and for the dysconjugacy ratio (vorDR).

		Impulse direction						
		Rightward			Leftward			
		Rightward dysconjugacy ratio	Eye				Leftward dysconjugacy ratio	
			Right	Left	Right	Left		
	Equation	AD_RE_/AB_LE_	AD_RE_	AB_LE_	AB_RE_	AD_LE_	AD_LE_/AB_RE_	
Gain Method	Regression	**1.12 (0.05)**	**1.08 (0.06)**	**0.97 (0.05)**	**0.94 (0.06)**	**1.07 (0.06)**	**1.14 (0.05)**	Mean (SD)
	Instantaneous	1.15 (0.08)	1.03 (0.09)	0.9 (0.09)	0.86 (0.08)	1.01 (0.08)	1.18 (0.07)	
	Median	1.10 (0.05)	1.06 (0.06)	0.96 (0.05)	0.94 (0.06)	1.06 (0.06)	1.13 (0.05)	
	Regression	**[1.02; 1.22]**	**[0.96; 1.2]**	**[0.87; 1.07]**	**[0.82; 1.06]**	**[0.95; 1.19]**	**[1.04; 1.24]**	Range
	Instantaneous	[0.99; 1.31]	[0.85; 1.21]	[0.72; 1.08]	[0.70; 1.02]	[0.85; 1.17]	[1.04; 1.32]	
	Median	[1.00; 1.2]	[0.94; 1.18]	[0.86; 1.06]	[0.82; 1.06]	[0.94; 1.18]	[1.03; 1.23]	
	Metric	Rightward_vorDR	VOR Gain				Leftward_vorDR	

Values for gains and ratios are reported as mean (SD). Normative ranges are reported with the lower and upper limits in parentheses. Values resulting from the calculation method with the best precision, regression gain, are highlighted in bold.

**Table 3:** Normative values and ranges for VOR asymmetry indices.

		VOR asymmetry index				
		Monocular		Binocular		
		Eye		Ductional VOR asymmetry index		
		Right	Left	AD_vorDAI	AB_vorDAI	
	Equation	|AD_RE_-AB_RE_|/AD_RE_+AB_RE_	|AD_LE_-AB_LE_|/AD_LE_+AB_LE_	|AD_RE_-AD_LE_|/AD_RE_+AD_LE_	|AB_RE_-AB_LE_|/AB_RE_+AB_LE_	
Gain Method	Regression	**7.0 (2.8)**	**4.5 (3.0)**	**1.3 (1.3)**	**2.2 (2.8)**	Median (IQR)
	Instantaneous	8.4 (3.8)	5.7 (4.0)	1.9 (1.9)	2.0 (3.8)	
	Median	5.6 (3.4)	4.8 (3.8)	2.0 (1.2)	1.6 (2.2)	
	Regression	**[3.4; 11.3]**	**[0.9; 11.0]**	**[0.0; 4.3]**	**[0.0; 6.1]**	Range
	Instantaneous	[4.3; 15.6]	[1.0; 17.2]	[0.0; 6.6]	[0.0; 8.5]	
	Median	[1.5; 13.4]	[0.5; 8.8]	[0.0; 4.9]	[0.0; 8.1]	
	Metric	m-vorAI [%]		vorDAI [%]		

Index values are reported as median with the interquartile range (IQR) in parentheses. Normative ranges are reported as percentile ranges from 2.5 to 97.5%, with lower and upper limits in parentheses. Index values resulting from the most precise gain calculation method, regression gain, are highlighted in bold.

The main effect found by the frequentist statistical analysis was a highly significant difference between ADduction and ABduction gains [*F*(1,38) = 350, *p* **<** 0.001]; the ADduction gains exceeded the ABduction gains (see Table 2 and Figure 5). The Bayes factors were BF_10_ > 10^17^, indicating “extreme evidence” for differences rather than equality. This finding holds for all three gain methods analyzed (see Table 2). Correspondingly, the monocular directional VOR asymmetry is also increased (see Table 3).

The regression and median gains analysis also showed significant differences between the levels of the eye (left, right) and repetition (test, retest) factors (*p* < 0.014), but the Bayes factors BF_10_ were < 7.5, indicating only “moderate evidence” for differences rather than equality. The regression and instantaneous gains also showed a significant interaction [*F*(1,38) = 12.85, *p* < 0.001] between the eye and duction factors, as shown in the interaction plots on the right of Figure 5. From these plots it can be concluded that the differences in ABduction gains between the left (0.97) and right (0.94) eyes, although significant, are small and clinically irrelevant compared to the main effect of duction.

It is noteworthy that the instantaneous gain of 0.95 (0.09), resulting from averaging over both eyes and duction directions, is comparable to the previously reported normal gain of 0.94 (0.1) from monocular vHIT recordings (5). However, the between-subject SD of the instantaneous gains of 0.09 was slightly lower than the standard deviations of 0.1 typically reported in the literature for normal vHIT gains (5). This may be due to the examiner’s 7 years of experience in administering the vHIT. The SDs of the regression and median gains of approximately 0.06 are considerably lower than the SDs of the instantaneous gain, reflecting a better inter-individual precision of these gain calculation methods.

We also calculated the averaged intra-individual SD as a measure of precision for the different gain calculation methods to answer the question of which metric should be used to report the results of future bvHIT examinations. Regression and median gain showed a significantly lower SD [*F*(77,77) = 1.46, *p* < 0.003] than the SD of instantaneous gain (0.02, 0.03, and 0.04, respectively).

The question of whether ABduction gain or ADduction gain is the more precise metric to assess VOR gain asymmetry was also addressed by analyzing the intra-individual SDs of the regression gains. Both duction directions showed SDs of 0.02, with no significant difference [*F*(77,77) = 1.46, *p* = 0.59]. Similarly, both ABduction and ADduction had comparable inter-individual SDs of 0.05. Therefore, in terms of precision, both directions of duction appear to be equally suitable for assessing VOR asymmetry. Similarly, the SDs for test and retest also showed the same values of 0.02 [*F*(77,77) = 1.46, *p* > 0.55], suggesting that no improvement in precision is to be expected from repeating a test. However, from test to retest, the pooled regression gain decreased slightly but significantly from 1.03 to 1.015 (0.06) [*F*(1,38) = 6.875, *p* = 0.013], possibly indicating an improvement in accuracy from retesting.

The repeatability coefficients for the three gain calculation methods are 0.06 for regression gain, 0.09 for instantaneous gain, and 0.07 for median gain. As regression gain was found to be the most precise metric, with the lowest values for both intra-and inter-individual SDs and repeatability coefficient, we recommend its use for future bvHIT gain reporting. Therefore, we focus our analysis and discussion on this metric. Accordingly, the regression gains are highlighted with bold letters in Tables 2, 3, which provide normative values and ranges.

#### Monocular VOR asymmetry

For regression gain, the median monocular VOR asymmetry recorded from one eye was 7.0% (IQR 2.8%) for the right eye and 4.5% (3.0%) for the left eye. The results reflect an ADduction-ABduction bias in monocular vHIT measurements, resulting in a directional gain preponderance (ADduction gains were always higher than ABduction gains in monocular recordings).

#### Ductional VOR asymmetry

We calculated ductional VOR asymmetry indices separately for ADduction and ABduction eye movement responses to leftward and rightward head impulses (Figures 3A,B). For the regression gain, the ADduction asymmetry index was 1.3% (IQR 1.3%) and the ABduction asymmetry was 2.2% (2.8%) (Table 3). These results indicate that ADduction asymmetry is less variable than ABduction asymmetry in the healthy subjects. Therefore, it provides a more precise assessment of peripheral vestibular function asymmetry. ADduction vHIT gains were not significantly different between the left and and right eyes, whereas ABduction vHIT gains were [*F*(1,38) = 12.85, *p* < 0.001] (Table 3 and interaction plot in Figure 5).

#### Binocular vHIT dysconjugacy ratio

The bvHIT dysconjugacy ratio (ADduction/ABduction) pooled for leftward and rightward head impulses was calculated as 1.08 (0.06) /0.95 (0.06). The resulting ratio of 1.13 (0.05) reflects the higher ADduction gains and should therefore be consistently greater than 1. Accordingly, the normative range, calculated as mean + -2xSD, is from 1.03 to 1.23. The dysconjugacy ratio is calculated separately for leftward and rightward impulses to assess the dysconjugacy during leftward or rightward VOR-induced eye movements. This would allow, for example, to identify unilateral or bilateral central oculomotor lesions.

## Discussion

We report normative ranges for the horizontal binocular video head impulse test (bvHIT) in 39 healthy participants aged 20 to 70 years.

### Difference between adducting and abducting eye VOR gains

Our results are consistent with a previous study using a gold standard scleral search coil to measure binocular eye movement responses to horizontal head impulse testing. In both studies, adduction gains exceeded those of abduction, resulting in directional gain asymmetry when recorded from only one eye (6).

### Mechanistic explanation of adduction delay with higher velocities during HIT

#### Different synaptic arcs

In the scleral search coil study (6), longer latencies were observed in the adducting eye but steeper velocity slopes than in the abducting eye. These have been interpreted as a result of the synaptic delay with a longer trisynaptic pathway and the different firing characteristics of the additional abducens internuclear neuron for adduction (1, 12, 13). The central neural pathways connecting the two horizontal semicircular canals to the recti eye muscles to mediate the horizontal VOR have been described in detail elsewhere (8).

#### Vergence system influence

The vergence system could also shape the differences between the velocity trajectories of the adducting and abducting eyes. Distance to the visual target is known to modulate the VOR gain, presumably via the vergence system (3–5). Conjugate gaze shifts between two distant targets at optical infinity, which require both eyes to rotate around the same angle, have been assumed to be driven solely by the conjugate subsystem. However, more recent studies have shown that such saccades are consistently accompanied by transient intrasaccadic vergence movements (the eyes initially diverge and then subsequently converge) resulting from dynamic asymmetries in the right and left eye movements (14).

#### Differences in muscle forces

There is evidence in the literature that the maximum active force of the medial rectus muscle responsible for adduction is approximately 25% greater than that of the lateral rectus muscle, which is responsible for abduction (15). The adduction force can be supported by a vergence command and by the tertiary muscle actions innervated by the same third cranial nerve. The abduction (sixth cranial nerve) can be supported by additive tertiary muscle actions innervated by the fourth and third cranial nerves.

### Our study

Our study showed partially similar results to the scleral search coil study with higher adduction gains compared to abduction gains of VOR eye movement responses to head impulse testing, resulting in a monocular VOR directional gain asymmetry. The binocular vHIT device used reflected this ADduction pattern in 100% of the recorded regression gains.

### Contribution to the field

#### bVHIT VOR asymmetry

Our data support the need to simultaneously record and compare vestibulo-oculomotor responses from both eyes during one impulse to obtain a more accurate vHIT asymmetry measure by comparing only adduction or only abduction gains of both eyes. This approach would avoid the preponderance of adduction over abduction, which is the cause of VOR asymmetry in monocular vHIT (6, 7). The previous search coil study (6) showed less variability in abduction gains. Our study showed similar within-subject and between-subject SD in both abduction and adduction. Considering that the abducting gains reflect a shorter three-neuron reflex arc with possibly less neural processing than in the four-neuron reflex arc of the adducting gains, we recommend the use of abduction for the assessment of VOR asymmetry (between the two eyes during abduction).

#### bvHIT gaze conjugacy

An additional advantage of the bvHIT is the assessment of gaze conjugacy during head impulse testing as a potential innovation in oculomotor assessment in otoneurology patients suffering from balance complaints accompanied by oculomotor disturbance. Therefore, we established a normative bvHIT dysconjugacy ratio (vorDR) dataset to describe the eye movement patterns during head impulse testing. Based on our normative data, the bvHIT dysconjugacy ratio should be in the range of 1.03 to 1.23 for regression gain. Thus, a vorDR of 1 or less could reflect adduction weakness, whereas a vorDR greater than 1.24 could be present in an abduction deficit.

The bvHIT dysconjugacy ratio can prove useful in supporting challenging diagnoses of dysconjugate eye movement disorders that may be accompanied by symptomatic diplopia or blurred vision. Loss of conjugacy during horizontal eye movements is a common and useful clinical sign of lateral or medial muscle palsies or weaknesses in different conditions such as stroke, in diabetic patients, internuclear ophthalmoplegia due to multiple sclerosis or stroke, Gradenigo syndrome in petrosal apicitis, intracranial hypertension, one and a half syndrome, and other ophthalmoplegias due to stroke or myasthenia gravis (16–18). The vorDR calculation is based on the direction of an impulse, which allows the assessment of both unidirectional (unilateral muscle palsies) or bidirectional dysconjugacy (such as bilateral INO).

### Artifacts

Mechanical factors, such as the translation of the adducting eyeball by pulling on the skin or the inertia of the eyeball itself, have been tested previously and are well addressed in the search coil study (6).

## Conclusion

Our study provides normative values for binocular vHIT (bvHIT) in healthy subjects. The ADducting eye has a higher vHIT gain than the ABducting eye. This AD-AB preponderance causes a directional gain bias in monocular vHIT. The binocular bvHIT measurement eliminates this bias by comparing the VOR gains of the abduction-only or the adduction-only movements of both eyes.

We also provide a novel bvHIT dysconjugacy ratio that adds a new advantage to vHIT testing: an assessment of inter- and infra-nuclear vestibulo-oculomotor central pathways and muscle action to vHIT. The dysconjugacy ratio reflects the action of horizontal gaze-yoked muscles and associated synaptic arcs. In conclusion, the main advantages of binocular bvHIT over monocular vHIT are, on the one hand, a more accurate measurement of vHIT gain asymmetry by an analysis of ductional VOR gains, and, on the other hand, an additional oculomotor assessment by evaluation of gaze conjugacy during head impulse testing.

## Data availability statement

The raw data supporting the conclusions of this article will be made available by the authors, without undue reservation.

## Ethics statement

The study was performed according to the ethical standards of the Declaration of Helsinki and following procedure approval of the Ethics Committee of the University Hospital Hradec Kralove (Reference number 202106 P08). The patients/participants provided their written informed consent to participate in this study. Written informed consent was obtained from the individual(s) for the publication of any identifiable images or data included in this article.

## Author contributions

MS designed the study. MS and ES conducted the study and measurements and wrote the manuscript. ES and TS provided statistical analysis. MC, VC, OP, JK, and MV provided critical revisions to the draft and read and approved the final manuscript.

## Funding

This study was partially supported by grant projects of the Ministry of Health of the Czech Republic (FN HK 00179906) and of the Charles University in Prague, by the Cooperatio Program, research area NEUR and area SURG, Faculty of Medicine in Hradec Kralove and 3rd Faculty of Medicine, Prague.

## Conflict of interest

ES is the general manager and a shareholder of EyeSeeTec GmbH.

The remaining authors declare that the research was conducted in the absence of any commercial or financial relationships that could be construed as a potential conflict of interest.

## Publisher’s note

All claims expressed in this article are solely those of the authors and do not necessarily represent those of their affiliated organizations, or those of the publisher, the editors and the reviewers. Any product that may be evaluated in this article, or claim that may be made by its manufacturer, is not guaranteed or endorsed by the publisher.
